# Attitudes to Cardiopulmonary Resuscitation and Defibrillator Use: A Survey of UK Adults in 2017

**DOI:** 10.1161/JAHA.117.008267

**Published:** 2019-03-28

**Authors:** Claire A. Hawkes, Terry P. Brown, Scott Booth, Rachael T. Fothergill, Niroshan Siriwardena, Sana Zakaria, Sara Askew, Julia Williams, Nigel Rees, Chen Ji, Gavin D. Perkins

**Affiliations:** ^1^ Warwick Clinical Trials Unit Warwick Medical School University of Warwick Coventry United Kingdom; ^2^ Intensive Care Medicine University Hospitals Birmingham NHS Foundation Trust Birmingham United Kingdom; ^3^ Clinical Audit and Research London Ambulance Service NHS Trust London United Kingdom; ^4^ School of Health and Social Care University of Lincoln United Kingdom; ^5^ Healthcare Innovation Directorate British Heart Foundation London United Kingdom; ^6^ Research and Development Department South East Coast Ambulance Service NHS Foundation Trust Crawley United Kingdom; ^7^ School of Health and Social Work;University of Hertfordshire Hatfield United Kingdom; ^8^ Welsh Ambulance Service NHS Trust Research and Innovation Institute of Life Science Swansea University Wales United Kingdom; ^9^ Strategy and International Affairs British Heart Foundation London United Kingdom

**Keywords:** cardiac arrest, education, education campaigns, out‐of‐hospital cardiac arrest, prehospital care, resuscitation, Cardiopulmonary Arrest, Cardiopulmonary Resuscitation and Emergency Cardiac Care

## Abstract

**Background:**

Bystander cardiopulmonary resuscitation (CPR) and public access defibrillator (PAD) use can save the lives of people who experience out‐of‐hospital cardiac arrest. Little is known about the proportions of UK adults trained, their characteristics and willingness to act if witnessing an out‐of‐hospital cardiac arrest, or the public's knowledge regarding where the nearest PAD is located.

**Methods and Results:**

An online survey was administered by YouGov to a nonprobabilistic purposive sample of UK adults, achieving 2084 participants, from a panel that was matched to be representative of the population. We used descriptive statistics and multivariate logistic regression modeling for analysis. Almost 52% were women, 61% were aged <55 years, and 19% had witnessed an out‐of‐hospital cardiac arrest. Proportions ever trained were 57% in chest‐compression‐only CPR, 59% in CPR, and 19.4% in PAD use. Most with training in any resuscitation technique had trained at work (54.7%). Compared with people not trained, those trained in PAD use said they were more likely to use one (odds ratio: 2.61), and those trained in CPR or chest‐compression‐only CPR were more likely to perform it (odds ratio: 5.39). Characteristics associated with being trained in any resuscitation technique included youth, female sex, higher social grade, and full‐time employment.

**Conclusions:**

In the United Kingdom, training makes a difference in people's willingness to act in the event of a cardiac arrest. Although there is considerable opportunity to increase the proportion of the general population trained in CPR, consideration should be also given to encouraging training in PAD use and targeting training for those who are older or from lower social grades.

**Clinical Perspective**

**What Is New?**

- In a representative survey of 2084 adults from the United Kingdom, 19% reported having witnessed an out‐of‐hospital cardiac arrest; 60% reported training in cardiopulmonary resuscitation techniques at some time, but only 27% in the past 5 years; and only 20% had ever trained in public access defibrillator use.
- Being younger, employed, and from a higher social grade were positively associated with having trained in cardiopulmonary resuscitation techniques.

**What Are the Clinical Implications?**

- There is potential in the United Kingdom to encourage regular retraining in cardiopulmonary resuscitation, to increase public access defibrillator training rates, and to target training to groups of people who are older, from lower social grades, or those not in the workforce.

## Introduction

Apart from calling emergency medical services (EMS), cardiopulmonary resuscitation (CPR) is the most important bystander action to improve the chance of survival of someone having an out‐of‐hospital cardiac arrest (OHCA).^1^ In 2014, in England, bystanders witnessed 35% of OHCAs treated by EMS and administered bystander CPR in 61% of those events. The rate of bystander CPR for non–EMS‐treated witnessed OHCA (ie, those cases witnessed by bystanders and unwitnessed cases combined) was 55%.^2^ In Scotland, the rate may be between 40% and 50%, although EMS recording challenges suggest that bystander CPR rates may be underreported.^3^ The best performing EMS services report bystander CPR rates of 66% (Netherlands),^4^ 68.8% (Victoria, Australia),^5^ 69% (King County [Seattle], Washington, USA),^6^ and 73% (Norway).^7^

Use of an automated external defibrillator (AED), known as a public access defibrillator (PAD) when located in a public place, can further increase survival rates of OHCA patients presenting with a shockable rhythm (ventricular fibrillation or pulseless ventricular tachycardia).^8^, ^9^ In Scotland, the initial shockable rhythm rate was 25.1%^3^. In England, it was 20.6% of all 2014 OHCA cases^2^; however, only 2.4% of non–EMS‐treated witnessed cases had a PAD applied. Survival is higher for these patients than for those presenting with nonshockable rhythm. A US population–based cohort study reported survival rates of 38% in patients with a shockable rhythm compared with 9% in those without.^8^

In the United Kingdom, like many countries, initiatives and campaigns aim to increase numbers in the general population trained in CPR and to improve access to PADs, for example, the British Heart Foundation's (BHF's) Heartstart training courses, their Call Push Rescue training initiative, and the national Restart a Heart campaign.^10^ In a 2014 BHF survey, 47% of UK adults^11^ reported having trained in CPR at some time. Having skills is clearly important, but being willing to use them in an emergency situation will make a difference. The 2014 survey reported that 23% felt they would always perform CPR in an emergency situation (BHF cardiac arrest survey, unpublished data, 2014). Commonly reported reasons for not performing CPR are being afraid of doing more harm than good or lacking skills and knowledge (BHF cardiac arrest survey, unpublished data, 2014).^12^, ^13^ Training is important because it is known to improve confidence.^14^

Little is known about the proportion of the UK population that has ever witnessed an OHCA or the proportion that would need training to improve bystander CPR and PAD use. In other countries, increasing the proportion of the population trained has been associated with increased bystander CPR and survival rates.^15^ In King County, Washington, 79% of the population had attended CPR training,^16^ >32% more than in the United Kingdom in 2014. In addition, 40% reported confidence in their ability to perform CPR in an emergency, a proportion similar to that reported in the UK survey (BHF cardiac arrest survey, unpublished data, 2014). King County has one of the highest OHCA survival rates in the world.^6^

In the United Kingdom as a whole, little is known about the characteristics of those trained or their willing to act when witnessing an OHCA. Such information is useful to training providers, campaigners, policy makers, and researchers for identifying coverage and reach of training campaigns. In Scotland, 52% of adults reported having trained in CPR.^17^ Elsewhere, characteristics significantly associated with confidence to perform CPR were younger age, male sex, length of time since last training, and number of times trained.^16^, ^17^ Sex, age group, marital status, education level, employment, length of time since last training, and number of times trained were associated with greater likelihood of performing CPR, as was recent CPR training.^17^, ^18^

We conducted a survey of UK adults to determine the proportion of the population that had witnessed an OHCA, had trained in CPR and/or PAD use, was willing to perform CPR or use a PAD, and knew where their nearest PAD was. We investigated characteristics associated with being trained and willingness to act in the event of witnessing a cardiac arrest.

## Methods

### Data Sharing Statement

The data are not currently available to other researchers because of our agreement with our research partner.

Questions designed to meet the study aims were inserted into an online omnibus survey conducted by YouGov, a market research company. We worked with YouGov to optimize question clarity of meaning and ease of understanding.

### Sample

A sample of 2084 adults was achieved through YouGov's nonprobabilistic active sampling method. YouGov sampled from its panel of >360,000 adults registered and incentivized to participate in surveys. The achieved sample is weighted to be representative of UK adults in terms of age, sex, social class, and type of newspaper chosen (survey design variables).^19^

The YouGov sampling method is essentially a purposive quota.^19^ The sample is taken from an existing panel and randomly targets panelists with particular characteristics to achieve quotas that match the proportions of people with those characteristics in the UK census data. Weights can be added during analysis to ensure that the achieved sample is representative if the quotas differ from the target. The YouGov method addresses the risk of bias in the achieved sample by using a reliable data source as a frame (ie, the UK census) to determine both the quotas and the weights used in the analysis. This approach produces a representative sample of the UK adult population.

### Ethical Considerations

The University of Warwick's Biomedical and Scientific Research Ethics Committee approved the study (Ref REGO‐2016‐1906). Informed consent was presumed in those who chose to complete the survey, having read introductory information on its content and purpose.

### Survey Questions

Questions addressed witnessing an OHCA, receiving chest‐compression‐only CPR (CO‐CPR), training in CPR or defibrillator use, location of training, knowledge of nearest PAD location, and willingness to act on witnessing an OHCA (Data S1).

### Data Collection

Data were collected on 1 day in May 2017 via an online survey. Participants identified for the sample were sent an email with a survey link. YouGov returned the anonymized data set to the Out of Hospital Cardiac Arrest Outcomes project team for analysis.

### Data Analysis

We used descriptive statistics to describe the sample by sex, age, governmental geographical region, marital status, number of children in the household, and social media use. Social grade was categorized using the National Readership Survey classification system^20^ (A: workers in high managerial, administrative, or professional jobs; B: workers in intermediate managerial, administrative, or professional jobs; C1: workers in supervisory, clerical, and junior managerial, administrative, or professional jobs; C2: skilled manual workers; D: semi‐ and unskilled manual workers; E: state pensioners, casual or lowest grade workers, and those unemployed with state benefits only). We present the number of respondents and the weighted and unweighted percentages in Tables 1, 2, 3, 4, 5. Weighted percentages are used in the text. Differences in participant characteristics were compared for outcomes using the Pearson χ^2^ statistic.

Logistic regression was undertaken on the weighted data to examine the influence of demographic characteristics, whether a participant had ever witnessed an OHCA, and whether people had trained in CPR (combining any training in either or both CO‐CPR and CPR) or AED use. In addition, these factors and training characteristics were assessed for their impact on willingness to call EMS, perform CO‐CPR, perform CPR, go and get a PAD, and use a PAD. Initially, single‐variable regression models were developed; any variable with *P*<0.2 in the bivariate analysis was integrated into the final model. A backward stepwise regression model was then developed, using a significance level of *P*>0.05 for removal from the model. Inclusion of weights and survey design variables can increase the risk of collinearity, resulting in less precise model estimates with larger standard errors.^21^ The existence of collinearity inflates the variances of the parameter estimates and, consequently, can cause incorrect inferences about relationships between explanatory and response variables. We tested for collinearity by calculating the variance inflation factor. If the variance inflation factor for a variable in each model was >10, then that variable was removed from the model.

All analyses were conducted using Stata/SE 15.1 (StataCorp).

## Results

### Demographic Characteristics

Table 1 presents the characteristics of the 2084 participants. It also shows demographics of participants who had indicated they had witnessed a cardiac arrest (18.8%). More than half were women (51.5%), 55.5% had full‐ or part‐time work, and 24.1% were retired. Most lived in a household without children (72.9%). More than half the sample (57%) was in social grade A, B, or C1. Nearly 60% were married, living as married, or in a civil partnership. About 81% of participants had never witnessed an OHCA. As expected, a greater proportion of older individuals had witnessed an OHCA. In addition, significantly more men, those in full‐time employment, and those living with a partner indicated they had witnessed an OHCA. There was no significant difference by social grade. The geographical spread of the sample is shown in Table S1 and includes a comparison with governmental 2017 midyear population estimates^22^ to show how close the achieved sample was to this population characteristic.

**Table 1:** Demographic Characteristics of the Sample

Characteristic	Total Respondents (N=2084)	Weighted % (unweighted %)	Witnessed a Cardiac Arrest, n (%)				
			No		Yes		χ^2^ (*P* Value)
			n (n=1688)	Weighted, % (Unweighted, %)	n (n=396)	Weighted, % (Unweighted, %)	
Age group, y							
18–24	252	11.5 (12.1)	220	87.3 (87.3)	32	12.7 (12.7)	13.61 (0.009)
25–34	315	16.5 (15.1)	254	81.0 (80.6)	61	19.0 (19.4)	
35–44	339	17.0 (16.3)	287	84.9 (84.7)	52	15.1 (15.3)	
45–54	340	15.9 (16.3)	269	79.1 (79.1)	71	20.9 (20.9)	
≥55	838	39.1 (40.2)	658	78.7 (78.5)	180	21.3 (21.5)	
Sex							
Female	1121	51.5 (53.8)	927	82.6 (82.7)	194	17.4 (17.3)	4.53 (0.033)
Male	963	48.5 (46.2)	761	79.7 (79.0)	202	20.3 (21.0)	
Number of children in household							
None	1532	72.9 (73.5)	1256	82.3 (82.0)	276	17.8 (18.0)	4.71 (0.095)
≥1	379	20.2 (18.2)	301	79.4 (79.4)	78	20.6 (20.6)	
Unknown	173	6.9 (8.3)	131	76.1 (75.7)	42	23.9 (24.3)	
Work status							
Full‐time (≥30 h)	816	39.9 (39.2)	641	78.5 (78.6)	175	21.5 (21.4)	23.4 (0.001)
Part‐time (<30 h)	312	15.6 (15.0)	268	86.4 (85.9)	44	13.0 (13.6)	
Retired	513	24.1 (24.6)	407	79.6 (79.3)	106	20.4 (20.7)	
Unemployed	71	3.5 (3.4)	59	84.0 (83.1)	12	16.0 (16.9)	
Not working	166	8.0 (8.0)	136	83.0 (81.9)	30	17.0 (18.1)	
Full‐time student	149	6.7 (7.1)	136	91.2 (91.3)	13	8.8 (8.7)	
Unknown	57	2.8 (2.7)	41	72.4 (71.9)	16	27.6 (28.1)	
Social grade^a^							
A	275	12.4 (13.2)	220	79.8 (80.0)	55	20.2 (20.0)	1.87 (0.867)
B	345	15.6 (16.6)	272	79.0 (78.8)	73	21.0 (21.2)	
C1	654	29.0 (31.4)	535	81.6 (81.8)	119	18.4 (18.2)	
C2	344	21.0 (16.5)	283	83.2 (82.3)	61	16.8 (17.7)	
D	224	10.7 (10.7)	182	80.7 (81.3)	42	19.3 (18.7)	
E	242	11.3 (11.6)	196	81.3 (81.0)	46	18.7 (19.0)	
Marital status							
Married	964	45.9 (46.3)	762	79.0 (79.0)	202	21.0 (21.0)	14.96 (0.021)
Living as married	259	12.6 (12.4)	208	81.1 (80.3)	51	18.9 (19.7)	
Separated	31	1.5 (1.5)	27	87.9 (87.1)	4	12.1 (12.9)	
Divorced	133	6.2 (6.4)	116	87.6 (87.2)	17	12.4 (12.8)	
Widowed	74	3.5 (3.6)	53	72.1 (71.6)	21	27.9 (28.4)	
Never married	598	29.0 (28.7)	503	84.3 (84.1)	95	15.7 (15.9)	
Civil partnership	25	1.3 (1.2)	19	75.0 (76.0)	6	25.0 (24.0)	
Used social messaging in past 30 d							
Yes	1566	75.3 (75.1)	1260	80.7 (80.5)	306	19.3 (19.5)	1.19 (0.276)
No	518	24.7 (24.9)	428	82.6 (82.6)	90	17.4 (17.4)	
Use social media networks in past 30 d							
Yes	1667	81.0 (80.0)	1345	81.0 (80.7)	322	19.0 (19.3)	0.53 (0.465)
No	417	19.0 (20.0)	343	82.0 (82.3)	74	18.0 (17.7)	

a A: high managerial, administrative, or professional (4% of population January–December 2016); B: intermediate managerial, administrative, or professional (23%); C1: supervisory, clerical, and junior managerial, administrative, or professional (28%); C2: skilled manual worker (20%); D: semi‐ and unskilled manual worker (15%); E: state pensioner, casual or lowest grade worker, unemployed with state benefits only (10%).^20^

### Resuscitation Training

Table 2 details the type of resuscitation training and how recently this had been undertaken. Almost 57% had been trained in CO‐CPR, but only ≈27% within the past 5 years. A similar proportion also reported receiving CPR training (59%), and again only ≈27% received it within the past 5 years. About 97% of those trained in CO‐CPR had also trained in CPR, 3% had trained only in CO‐CPR. Just >40% had never been trained in CPR. Almost 80% (78.4%) had never been trained in defibrillator use.

**Table 2:** Type of Resuscitation Training Received and How Recently

Type of Resuscitation Training/When Most Recently Trained	n	Weighted, % (Unweighted, %)
CO‐CPR		
≤1 y	217	10.5 (10.4)
>1 y ago but within the past 5 y	345	16.7 (16.6)
>5 y ago but within the past 10 y	212	9.9 (10.2)
>10 y ago	414	19.6 (19.9)
Never trained	840	40.3 (40.3)
Don't know/can't recall	56	2.9 (2.7)
CPR		
≤1 y	213	10.4 (10.2)
>1 y ago but within the past 5 y	351	17.0 (16.8)
>5 y ago but within the past 10 y	219	10.2 (10.5)
>10 y ago	446	21.1 (21.4)
Never trained	803	38.6 (38.5)
Don't know/can't recall	52	2.9 (2.5)
Defibrillator use		
≤1 y	157	7.5 (7.5)
>1 y ago but within the past 5 y	145	7.0 (7.0)
>5 y ago but within the past 10 y	56	2.6 (2.7)
>10 y ago	48	2.3 (2.3)
Never trained	1636	78.4 (78.5)
Don't know/can't recall	42	2.2 (2.0)

CO‐CPR indicates chest‐compression‐only cardiopulmonary resuscitation; CPR, cardiopulmonary resuscitation (compression and mouth‐to‐mouth).

Table 3 shows the likelihood of training in the various resuscitation techniques comparing those who reported they had and had not witnessed an OHCA. Those who had witnessed one were significantly more likely to have trained previously in a resuscitation technique (any or all of CO‐CPR, CPR or AED use; *p*<0.001).

**Table 3:** Likelihood of Training in Resuscitation Skills by Having Witnessed OHCA or Not

	Witnessed OHCA				χ^2^ (*P* Value)
	Yes (n=396)		No (n=1688)		
	n	Weighted, % (Unweighted, %)	n	Weighted, % (Unweighted, %)	
Ever trained in any resuscitation skill					
Yes	304	76.6 (76.8)	963	56.7 (57.1)	52.3
No	92	23.4 (23.2)	725	43.3 (42.9)	(<0.001)
Ever trained in any resuscitation skills in the past 5 y					
Yes	174	44.6 (43.9)	434	25.9 (25.7)	51.6
No	222	55.4 (56.1)	1254	74.1 (74.3)	(<0.001)
Ever trained in CPR (chest compressions and mouth‐to‐mouth)					
Yes	304	76.6 (76.7)	958	56.4 (56.8)	53.8
No	92	23.4 (23.2)	730	43.6 (43.2)	(<0.001)
Ever trained in CPR (chest compressions and mouth‐to‐mouth) in the past 5 years					
Yes	170	43.7 (42.9)	425	25.4 (25.2)	49.5
No	226	56.3 (57.1)	1263	74.6 (74.8)	(<0.001)
Ever trained in defibrillator use					
Yes	140	35.3 (35.4)	266	15.7 (15.8)	78.5
No	256	64.7 (64.6)	1422	84.3 (84.2)	(<0.001)
Trained in defibrillator use in the past 5 y					
Yes	108	27.5 (27.3)	194	11.5 (11.5)	64.5
No	288	72.5 (72.7)	1494	88.5 (88.5)	(<0.001)

CPR indicates cardiopulmonary resuscitation (compression and mouth‐to‐mouth); OHCA, out‐of‐hospital cardiac arrest.

Of those who had ever been trained in ≥1 resuscitation technique (n=1267), more than half (54.7%) had been trained at work (Table 4). In addition 15% trained while at school, and almost 12% trained at a youth organization, such as scouts.

**Table 4:** Location of Most Recent Training in ≥1 of CO‐CPR, CPR, or Defibrillator Use (n=1267)

Location of Training	n	Weighted, % (Unweighted, %)
At work	685	54.7 (54.1)
At school while a student	194	15.0 (15.3)
At a youth organization	150	11.8 (11.8)
At a school or other community building as an adult (not as a student)^a^	175	13.6 (13.8)
At a local event^b^	38	3.0 (3.0)
Watching a video/film online/offline	29	2.4 (2.3)
Watching a television program online/offline	31	2.3 (2.4)
A relative/someone I know showed me what to do after they had been trained in CPR	25	1.9 (2.0)
On a computer reading websites	11	0.9 (0.9)
Via an app	4	0.3 (0.3)
Other	183	14.2 (14.4)
Don't know/can't recall	16	1.2 (1.3)

CO‐CPR indicates chest‐compression‐only cardiopulmonary resuscitation; CPR, cardiopulmonary resuscitation (compression and mouth‐to‐mouth).

a For example, a village or community hall.

b For example, county or village show, ambulance station open day.

### Likelihood of Acting

Most participants (92.9%) said they would very likely or fairly likely phone EMS if they witnessed an OHCA (Table 5). More than half were very likely or fairly likely to perform CO‐CPR (55.6%) and CPR (57.6%). Rates rose to 70.0% and 76.5%, respectively, for those who had received any type of resuscitation training.

**Table 5:** Likelihood of Acting on Witnessing a Cardiac Arrest by Having Witnessed OHCA or Not

	Total (N=2084)		Witnessed OHCA			
			Yes (n=396)		No (n=1688)	
	n	Weighted, % (Unweighted, %)	n	Weighted, % (Unweighted, %)	n	Weighted, % (Unweighted, %)
Call EMS						
Yes	1942	92.9 (93.2)	379	95.8 (95.7)	1563	92.2 (92.6)
No	142	7.1 (6.8)	17	4.2 (4.3)	125	7.8 (7.4)
CO‐CPR						
Yes	1159	55.6 (55.6)	268	68.2 (67.7)	891	52.6 (52.8)
No	925	44.4 (44.4)	128	31.8 (32.3)	797	47.4 (47.2)
Perform CPR						
Yes	1207	57.6 (57.9)	287	72.5 (72.5)	920	54.1 (54.5)
No	877	42.4 (42.1)	109	27.5 (27.5)	768	45.9 (45.5)
Go and get a defibrillator						
Yes	872	41.9 (41.8)	222	56.7 (56.1)	650	38.5 (38.5)
No	1212	58.1 (58.2)	174	43.3 (43.9)	1038	61.5 (61.5)
Use a defibrillator						
Yes	728	35.1 (34.9)	205	52.3 (51.8)	523	31.2 (31.0)
No	1356	64.9 (65.1)	191	47.7 (48.2)	1165	68.8 (69.0)

CO‐CPR indicates chest‐compression‐only cardiopulmonary resuscitation; CPR, cardiopulmonary resuscitation (compression and mouth‐to‐mouth); EMS, emergency medical services; OHCA, out‐of‐hospital cardiac arrest.

Just >40% of all participants (41.9%) were likely or very likely to go and get an AED, increasing to 52% for those who had any form of resuscitation training. For using an AED, rates were 35.1% and 46.6%, respectively. For those trained in defibrillator use, the figures rose to >78% for both willingness to get an AED and to use one.

Those who had witnessed an OHCA said they were more likely than those who had not witnessed one to act on witnessing an OHCA. Table 5 gives details by type of action.

### Location of Nearest PAD to Home

More than half (56.0%) of all participants did not know where the nearest PAD was to their home. Almost 21% (20.6%) reported there was a PAD >1 km from their home, and 11.4% reported it was 500 m to 1 km away, 6.2% reported it was 200 to 500 m distant, 3.3% reported it was 100 to 200 m away, and 2.5% said it was <100 m away.

### Characteristics of Those Willing to Intervene If Witnessing a Cardiac Arrest

Bivariate logistic regression analysis of willingness to act when witnessing an OHCA and whether people had trained in CPR (combining any training in either or both CO‐CPR and CPR) or AED use indicated significant associations with all participant characteristics. Backward stepwise regression of significant variables resulted in the final models in Tables 6 and 7. In the analysis, a number of the categories were combined for specific variables for which no statistical differences were observed between categories. Age groups 18 to 24 and 25 to 34 years were combined, as were 35 to 44, 45 to 64, and ≥55 years. The number of children in the household was categorized as *none* and *≥1/unknown*. Work status was divided into 3 categories: *full/part‐time, student*, and *other*. Social grade was divided into 2 groups: A, B, and C1 and C2, D, and E. Marital status was split into 2 categories: *married/living as married* and *other*.

**Table 6:** Estimated Predictors in Final Regression Models for Willingness to Call EMS, Perform CPR, and Get and Use a Defibrillator

Variable	Willingness, OR (95% CI)				
	To Call EMS	To Perform CO‐CPR	To Perform CPR	To Go and Get Defibrillator	To Use a Defibrillator
Sex (female)				0.80 (0.66–0.97)	0.63 (0.51–0.78)
Married or living as married		1.30 (1.07–1.57)	1.35 (1.10–1.66	1.40 (1.10–1.80	
No children in household	0.60 (0.40–0.90)	0.89 (0.82–0.96)			
Aged 18–34 y	0.46 (0.30–0.69)				
Social grade A, B, or C1^a^	1.70 (1.17–2.47)				
Use of social media in past 30 d	1.85 (1.21–2.83)				
Witnessed OHCA		1.41 (1.10–1.82)	1.53 (1.17–2.01)		1.61 (1.23–2.12)
Ever trained		3.39 (2.72–4.23)			
Ever trained in past 5 y		1.80 (1.40–2.31)	2.33 (1.76–3.07)		
Ever trained in CPR (CO‐CPR and/or CPR)	9.18 (4.39–19.23)		5.39 (4.29–6.76)	1.67 (1.33–2.10)	2.14 (1.69–2.72)
Ever trained in CPR (CO‐CPR and/or CPR) in past 5 y				1.37 (1.04–1.80)	
Ever trained in defibrillator use	0.31 (0.11–0.82)			2.62 (1.71–4.01)	2.64 (1.71–4.08)
Ever trained in defibrillator use in past 5 y	5.96 (1.61–22.15)			2.26 (1.32–3.89)	5.20 (3.07–8.82)

CO‐CPR indicates chest‐compression‐only cardiopulmonary resuscitation; CPR, cardiopulmonary resuscitation (compression and mouth‐to‐mouth); EMS, emergency medical services; OHCA, out‐of‐hospital cardiac arrest; OR, odds ratio.

a A: high managerial, administrative, or professional (4% of population, January–December 2016); B: intermediate managerial, administrative, or professional (23%); C1: supervisory, clerical, and junior managerial, administrative, or professional (28%); C2: skilled manual worker (20%); D: semi‐ and unskilled manual worker (15%); E: state pensioner, casual or lowest grade worker, unemployed with state benefits only (10%).^20^

**Table 7:** Estimated Predictors in Final Regression Models for Types of Training

Variable	Ever Trained, OR (95% CI)					
	CPR (CO‐CPR and/or CPR) and/or Debrillator Use	CPR (CO‐CPR and/or CPR) and/or Debrillator Use in Past 5 y	CPR (CO‐CPR and/or CPR) Only	CPR (CO‐CPR and/or CPR) Only in Past 5 y	Debrillator Use	Debrillator Use in Past 5 y
Sex (female)	1.25 (1.04–1.50)		1.24 (1.03–1.50)			
Married or living as married	1.37 (1.13–1.66)		1.37 (1.13–1.66)			
No children		1.34 (1.07–1.67)		1.39 (1.11–1.74)		
Aged 18–34 y		1.63 (1.27–2.08)		1.63 (1.28–2.08)		1.37 (1.02–1.85)
Social grade A, B, or C1^a^	1.25 (1.03–1.51)		1.24 (1.02–1.50)			
Work status: working full/part‐time	1.57 (1.29–1.91)	3.25 (2.56–4.13)	1.55 (1.27–1.88)	3.22 (2.54–4.10)	2.27 (1.75–2.93)	3.33 (2.43–4.58)
Work status: full‐time student	2.39 (1.57–3.65)	3.51 (2.26–5.46)	2.33 (1.53–3.54)	3.51 (2.26–5.45)	1.94 (1.20–3.13)	2.34 (1.31–4.19)
Use of social media in past 30 d	1.29 (1.03–1.62)		1.31 (1.04–1.65)		1.49 (1.08–2.04)	1.51 (1.03–2.20)
Witnessed OHCA	2.60 (2.00–3.37)	2.62 (2.05–3.35)	2.62 (2.02–3.40)	2.58 (2.02–3.30)	3.06 (2.37–3.95)	3.14 (2.36–4.18)

CO‐CPR indicates chest‐compression‐only cardiopulmonary resuscitation; CPR, cardiopulmonary resuscitation (compression and mouth‐to‐mouth); EMS, emergency medical services; OHCA, out‐of‐hospital cardiac arrest; OR, odds ratio.

a A: high managerial, administrative, or professional (4% of population, January–December 2016); B: intermediate managerial, administrative, or professional (23%); C1: supervisory, clerical, and junior managerial, administrative, or professional (28%); C2: skilled manual worker (20%); D: semi‐ and unskilled manual worker (15%); E: state pensioner, casual or lowest grade worker, unemployed with state benets only (10%).^20^

Table 6 presents the final regression models for willingness to phone EMS, to perform CPR, to go and get and use a defibrillator. Although most people were willing to phone EMS if they witnessed an OHCA, the most significant positive factors in the model were ever having training in CPR and/or CO‐CPR (odds ratio [OR]: 9.2) or in defibrillator use within the past 5 years (OR: 6.0). Other characteristics associated with greater willingness to phone EMS were social grades A, B, and C1 (OR: 1.6) and recently using social media (OR: 1.85). Individuals aged 18 to 34 years, compared with those aged ≥35 years, and those who lived in households with no children compared with those who lived in households with children were less likely to phone EMS (OR: 0.46 and 0.60, respectively).

Training, especially if an individual had undertaken any training (OR: 3.4), was the most significant factor in the model investigating people's willingness to perform CO‐CPR. A person who said he or she had witnessed a cardiac arrest was ≈1.5 times more likely to say they would act than those who had not witnessed one. People who were married or living as married were also more willing to perform CO‐CPR (OR: 1.3). Having ever trained in CPR was the most important factor in the willingness of an individual to perform CPR (OR: 5.39), with ever having training in any resuscitation technique‐ in the past 5 years (OR: 2.33) being a significant factor. Having witnessed an arrest (OR: 1.53) and being married/living as married (OR: 1.35) were also predictive factors.

Training in defibrillator use ever (OR: 2.62) or in the past 5 years (OR: 2.26) and training in CPR (either CO‐CPR or CPR; OR for both] ever (OR: 1.67) or training in the past 5 years (OR: 1.37) were the most significant predictive factors for an individual's willingness to go and get or use an AED. Those who were married were more likely to go and get an AED (OR: 1.4). Those who had trained in defibrillator use in the past 5 years were >5 times more willing to use one (OR: 5.2) than those who had not. Having witnessed a cardiac arrest was also a predictor of willingness to use one (OR: 1.61). Women were less likely than men to go and get (OR: 0.8) or use (OR: 0.63) an AED.

### Characteristics of Those Who Had Received Training

Table 7 presents model parameters for types of training received. Having witnessed a cardiac arrest was a significant factor in whether people had ever trained in all types of resuscitation techniques ever (OR: 2.6) or in the past 5 years (OR: 2.26), as was having full‐ or part‐time work and being a full‐time student. Women were more likely to have trained in any type of resuscitation technique (OR: 1.25) and in any type of CPR (OR: 1.24). Similarly, being married/living as married was also a significant predictor for these types of training (OR: 1.37 and 1.24, respectively). The ORs for ever having trained in any resuscitation technique and ever having trained in CPR were also significantly greater for people in higher social grades (A, B, and C1; OR: 1.25 and 1.24, respectively) compared with those in the lower social grades (C2, D, and E). Having no children and being aged 18 to 34 years were both significant indicators of whether an individual had undergone any type of training and training in CPR, both in the past 5 years. Use of social media in the past 30 days was also associated with increased odds of ever training in CPR and defibrillator use.

## Discussion

This survey provides an overview of the UK public's experience of training in CPR techniques and attitudes toward performing them. Approximately 60% had undertaken some form of CPR training. However, these figures fell to 17% to 27% for those trained within the past 5 years. Less than 20% had trained in AED use. Those from lower social grades, those not working, and older people were less likely to have trained. The workplace was the most frequently reported place of training (55%) and just more than a quarter (27%) trained at school or in a youth organization. People who had witnessed an OHCA were 2.5 to 3 times more likely to have trained in any resuscitation technique than those who had never witnessed an OHCA.

Training had a positive effect on participants’ self‐reported willingness to act if they witnessed an OHCA, with almost three quarters of those trained in CPR or AED use saying they would likely perform CPR or use an AED. Having witnessed an OHCA meant participants were approximately 1.5 times more likely to report they were willing to perform CPR and use an AED than those who had not witnessed one.

Survival rates from OHCA of all etiologies in the United Kingdom are reported at 6%^3^ to 8%^2^ and are known to be lower than other countries’ best performing areas.^6^, ^7^, ^15^ Although reasons for OHCA survival are multifactorial and causal relationships cannot be established in observational studies, in other countries, increased bystander CPR and defibrillation rates have been linked to improved survival rates. An evaluation study of the Take Heart America program reported an increased survival rate and improvement of 9% in bystander CPR rates.^23^ In Norway, where there have been sustained initiatives to improve OHCA survival, CPR, and defibrillation rates for the past 2 decades, Lindner et al^7^ reported improvement in bystander CPR rates from 60% to 73% and in survival to discharge rates from 18% to 25% in OHCA cases with cardiac etiology. Ninety percent of Norwegian survey participants reported receiving first aid training, which likely (but not definitely) included CPR training.^24^ In Denmark, Wissenberg et al^15^ reported a positive association between increasing bystander CPR rates and survival rates, with both improving over 10 years. Such associations over the long term have not yet been reported in the United Kingdom.

The development of campaigns and national strategies to improve bystander CPR and defibrillator rates also have potential to improve UK OHCA survival rates. Overall, 19% of survey participants reported having witnessed a cardiac arrest. Training was associated in this study with greater willingness to perform CPR or defibrillation. Approximately 60% of participants had undertaken some CPR training. This compares favorably to 47% reported in 2014.^11^ In the United States, 65% of the population reported ever having trained in CPR.^25^

Retention of skills affects confidence to act. Although little is known about retention of CPR knowledge among members of the public, skill performance will decay over time.^26^ In this study, only 27% had trained within the past 5 years, compared with a Norwegian survey^24^ that reported 54% attending first aid training within the past 5 years and a US survey reporting 18% trained in CPR in the past 2 years.^25^

Less than 20% of our survey sample had been trained in AED use; however, the impact of training on willingness to get and use an AED was marked compared with people who had not been trained. PAD use in England was 2.4% in 2014,^2^ with similar rates in other countries.^15^ Initiatives to increase PAD availability and use could be improved, as 20.6% of OHCA patients in England^2^ and 25.1% in Scotland^3^ present with a shockable rhythm.

Across Europe between 46.4% and 79.9% of OHCAs are reported as occurring in the home,^27^ so we asked whether people knew where the PAD nearest to their home was located. Although the optimal distance from arrest location to PAD location is not yet established, within 200 to 500 m is suggested.^28^ However, only 5.7% reported a PAD was located within 200 m, and another 6.2% reported it within 500 m of their home. More work on increasing knowledge of PAD locations and the hours they are accessible is needed to enable targeted campaigns to improve their use and inform placement of new PADs.^28^ This may include consideration of placement in public places in predominantly residential locations, since the majority of OHCAs occur in people's homes.

More than half of this survey's participants received training in the workplace, suggesting it is a good place to target training events. However, 42% were not in the workforce, including 24% who were retired. Age was a significant factor in some of our models, with younger people more likely to have trained, especially within the past 5 years, than older people. Facilitators to access training for older people have been reported, such as increasing availability of training and using convenient locations and transport to help get there, advertising, providing information about CPR training, and promoting its benefits^13^.

Training as a school student or in a youth organization accounted for 27% of adults’ training in this survey and demonstrates the importance of these organizations in training initiatives. Increasing CPR rates have been associated with mandatory training in schools,^15^ and the World Health Organization–endorsed statement “Kids Save Lives” recommends that annual training from age 12 upward should be mandatory.^29^ Training in UK schools is not currently mandatory, although the government has proposed to make it so by 2020.

Having witnessed an OHCA was associated with a greater likelihood of taking up training in all aspects of resuscitation skills and in self‐reported willingness to use these skills. Although some surveys of participants in CPR training events have asked for reasons for attending, including knowing someone with cardiac disease,^30^, ^31^ we were unable to locate any that had asked about previously witnessing a cardiac arrest. Pane et al reported that only 5.6% of participants in a mass CPR training event in the United States gave their own, a relative's, or a friend's cardiac disease as a reason for attending the training.^30^

Social grade was also a significant factor in being trained in CPR and AED use. Those in professional, management, and skilled work were more likely to have trained. In the United States, being in a lower socioeconomic group was independently associated with being less likely to have trained in CPR.^25^ In England^32^ and Scotland,^3^ as in other countries,^3^, ^33^, ^34^ more deprived areas have increased incidence of OHCA and lower bystander CPR and survival rates. Targeted training initiatives to reach more people working in unskilled jobs and living in deprived neighborhoods should be considered. Understanding barriers and facilitators to uptake of training among these groups could inform such initiatives. Barriers to training in high‐risk neighborhoods in Columbus, Ohio, were accessibility, cost, and distance to training; time of day training is held and unfamiliar location of training; distrust of EMS, police, and fire services; lack of knowledge; language of training; literacy; and the weather.^12^ Whether these barriers translate to the United Kingdom is unknown and warrants further research.

Other associations between participant characteristics and training include that women were more likely to have trained than men within the past 5 years; that younger people were more likely to have trained than older people, as is the case in the United States^25^; and that those who were married/living as married were more likely to have trained than those who were not. More work to understand interactions between characteristics, such as work status or social grade with sex and age, or age with social media use, and subsequent research to understand more about barriers and facilitators to accessing training for men and older people could inform targeting of training.

### Limitations

The survey is a snapshot on 1 day of a representative sample of UK adults. Sample surveys risk containing bias and, in this method, the risk is minimized during sampling. In the perhaps more familiar, probabilistic sampling methods, nonresponse bias can be calculated and can inform the application of weights during analysis. This approach is not possible in the nonprobabilistic panel sampling method that YouGov uses, and the (unresolved) debate in the literature suggests it may be a limitation for this study.^35^ Although it does not achieve a probabilistic sample, advantages of this method include delivery of a prespecified sample size, the ability to sample where appropriate population sampling frames are difficult to source and/or contact details for potential participants are lacking, a short time frame for data collection, and lower cost than many other survey methods. Bias in this approach to survey sampling needs to be viewed differently because the risk of bias is minimized during sampling. Mercer et al suggest that the type of method in which the quotas are set using data from a reliable source (in this case the UK Census) and then adding weights, if necessary—again, based on a reliable data source during analysis—is stronger. This 2‐phase approach can be considered more robust in reducing the risk of a biased sample than simply achieving quotas according to population characteristics limited to age, sex, and social group.^36^ We report on weighted data, using the more robust 2‐phase approach. We have provided comparisons in Table 2 of the spread of the achieved unweighted sample by geographical region compared with the UK government midyear population estimate.^22^ Differences in the resulting percentages between the weighted and unweighted samples are minimal.

The benefits of this sampling method are that we were able to achieve our desired sample size quickly within the limited resources available to the study team.

The survey was not deigned to establish causal links. However, we report associations among participant characteristics, training, and willingness to act in the event of witnessing a cardiac arrest. We cannot be sure that the reported likelihood of acting would translate into actual action in a real‐life cardiac arrest situation.

Data accuracy is limited by the participants’ understanding of the questions. Although we attempted to provide definitions of *cardiac arrest* and other terms that would be understood by the layperson, it is possible participants did not fully understand when responding.

We provide data for the first time in the United Kingdom on topics such as witnessing an OHCA or going to get an AED, so it is not possible to compare our findings with previous data to confirm or refute the accuracy of these findings. The YouGov sampling strategy aims to provide a representative sample, but it is possible our sample was biased for an unknown reason toward those who had witnessed an OHCA. It is also possible some participants did not understand the question and the explanations of what a cardiac arrest is and thus may have answered erroneously.

Whether there has really been a 13% rise in the proportion of UK adults trained in CPR since 2014 is difficult to tell simply by comparing 2 data sets from 2 cross‐sectional surveys.

## Conclusions

Training was clearly positively associated with people's willingness to act in the event of witnessing a cardiac arrest. Rates of those trained in CPR in the United Kingdom seem to be rising but are not yet at levels reported in areas with the best survival rates. The proportion of people trained within the past 5 years is much lower, however, and, given that skills may deteriorate over time, consideration should be given to encouraging people to regularly refresh their skills and any associated strategies evaluated.

Increasing the use of PADs for people experiencing OHCA is essential to save more lives. Given the low percentage of PAD use in England, training more people in AED use and raising awareness of PAD locations are essential.

Increasing or even introducing mandatory CPR and AED training in schools deserves consideration, as about a quarter of adults reported school or youth organizations as the source of their most recent training. Workplace training is important, and campaigns to encourage it should continue. In addition, offering training in multiple venues is important, with >40% of the adult population not in the workforce. Work to understand barriers to access training and using this information to target training initiatives to those living in lower income households or in higher risk neighborhoods and to older people should also be considered.

## Sources of Funding

The study is supported by research grants from the British Heart Foundation and the Resuscitation Council (UK).

## Disclosures

Hawkes, Brown, Booth, Ji and Perkins are employed by the University of Warwick, which receives grants from the British Heart Foundation (BHF) and the Resuscitation Council (UK) for the conduct of the Out of Hospital Cardiac Arrest Outcomes project. Gavin Perkins is supported as UK National Institute for Health Research (NIHR) Senior Investigator and Director of Research for the Intensive Care Foundation. Sana Zakaria and Sara Askew are employed by the BHF. Rachael Fothergill is employed by the London Ambulance Service NHS Trust and is also an honorary research fellow at the University of Warwick and currently receives research funding from the NIHR and the BHF. Niroshan Siriwardena currently receives research funding from the NIHR, Health Foundation, and Galen Pharmaceuticals. The remaining authors have no disclosures to report.

## Supporting information


**Data S1.** Survey Questions
**Table S1.** Geographical Spread of the Sample By UK Country and for England Region (Unweighted and Weighted) Compared With Mid‐2017 Population Estimates
