# Supplementary material for: Attitudes to Cardiopulmonary Resuscitation and Defibrillator Use: A Survey of UK Adults in 2017
Source: J Am Heart Assoc. 2019 Mar 28;8(7):e008267. doi: 10.1161/JAHA.117.008267 (PMC6509714; doi:10.1161/JAHA.117.008267)
Supplement: Supplementary file 1 — Data S1. Survey Questions Table S1. Geographical Spread of the Sample By UK Country and for England Region (Unweighted and Weighted) Compared With Mid‐2017 Population Estimates [file JAH3-8-e008267-s001.pdf]

SUPPLEMENTAL MATERIAL

Data S1.

Survey Questions

1. For the following question, by cardiopulmonary resuscitation (CPR) we mean an emergency procedure which is/ should be performed on a person suffering a cardiac arrest; and it involves chest compressions to maintain circulation until an ambulance arrives and rescue breathing (i.e. mouth-to-mouth resuscitation).

Please do not include any incidents that have happened within a hospital or nursing home and/ or any incidents that you attended to as part of your job (e.g. as a doctor, nurse, paramedic).

Approximately, how many times in your life have you seen someone collapse and be in need of cardiopulmonary resuscitation (CPR)? (If you have never seen this please type "0" in the box below).

2. As a reminder, by cardiopulmonary resuscitation (CPR) we mean an emergency procedure which is/ should be performed on a person suffering a cardiac arrest; and it involves chest compressions to maintain circulation until an ambulance arrives and rescue breathing (i.e. mouth-to-mouth resuscitation).

For the following question, please think about the most recent time you have been trained in any of the following: cardiopulmonary resuscitation (CPR) or how to use a defibrillator (i.e. a machine that can give electric shocks to re-start the heart). This can be both formal (e.g. training you received from a medical professional, first aider etc.) and informal (e.g. self-taught, showed by a relative after they received training etc.) training.

Approximately, when, if **EVER**, was the most recent time you were trained in each of the following? (Please select one option on each row. If you have never been trained, please select the 'Not applicable' option)

	Within the last year	Longer than a year ago, but within the last 5 years	Longer than 5 years ago, but within the last 10 years	Longer than 10 years ago	Not applicable - I've never been trained in this	Don’t know/ can't recall
Chest compressions only (i.e. pressing up and down on the chest)						
Using a defibrillator (i.e. a machine which can deliver an electric shock to restart the heart)						
Chest compressions and rescue breathing (i.e. mouth-to-mouth resuscitation)						

Depending on participant response the survey automatically routed to Q3 if the participant had trained in anything, or routed to Q4 if they had never trained in anything

3. **You said you had been trained in; chest compressions (i.e. pressing up and down on the chest), chest compressions and mouth-to-mouth resuscitation, using a defibrillator...**

Through which, if any, of the following did you receive this training? (Please select all that apply)

- At school, whilst I was a pupil
- At a school or another community building (e.g. village hall, scout hut etc.) as an adult
- Via an app

- At work
- At scouts/ guides or another youth organisation
- A relative/ someone I know showed me what to do after they had been trained in CPR
- On a computer reading websites
- At a local event (e.g. county or village show, ambulance station open day etc.)
- Watching a television programme online/ offline
- Watching a video/ film online/ offline
- Other

4. For the following question, by "cardiac arrest" we mean when a person's heart stops beating and they stop breathing.

Please imagine that you were witnessing someone having a cardiac arrest in front of you...

Provided all of these options were available to you (i.e you had access to a phone, defibrillator etc.), which, if any, of the following would you be likely to do? (Please select all that apply)

	Very likely	Fairly likely	Not very likely	Not at all likely	Don't know
Go and get a publicly accessible defibrillator (i.e. a machine which can deliver an electric shock to restart the heart)					
Phone 999					

	Very likely	Fairly likely	Not very likely	Not at all likely	Don't know
Perform chest compressions and rescue breathing (i.e. mouth-to-mouth resuscitation)					
Perform chest compressions only					
Use a defibrillator (i.e. a machine which can deliver an electric shock to the restart the heart)					

5. As a reminder, for the following question by "defibrillator" we mean a machine which can deliver an electric shock to restart the heart.

Approximately how far do you think the nearest publicly accessible defibrillator is from your home? (Please select the option that best applies. If you don’t know, please select the Don't know option)

- Less than 100 metres
- At least 100 metres away, but less than 200 metres away
- At least 200 metres away, but less than 500 metres away
- At least 500 metres away, but less than a kilometre away
- More than one kilometre away
- Don't know

Table S1. Geographical spread of the sample by UK country and for England Region (unweighted and weighted) compared to mid-2017 population estimates.

Country/Region	CPR Survey (number, %)		UK mid-2017 population aged 16+y (number, %)*
	Unweighted	Weighted	
England	1,728 (82.7)	1750 (84)	44,234,600 (84.1)
North East	80 (3.8)	77 (3.7)	2,140,200 (4.1)
North West	232 (11.1)	237 (11.4)	5,761,500 (10.9)
Yorkshire & Humber	174 (8.3)	174 (8.4)	4,338,100 (8.2)
East Midlands	189 (9.1)	186 (8.9)	3,790,500 (7.2)
West Midlands	149 (7.1)	147 (7)	4,605,100 (8.8)
East of England	174 (8.3)	170 (8.1)	4,905,200 (9.3)
London	231 (11.1)	271 (13)	7,022,700 (13.3)
South East	313 (15.0)	306 (14.7)	7,203,100 (13.7)
South West	186 (8.9)	182 (8.7)	4,468,200 (8.5)
Wales	106 (5.1)	101 (4.8)	2,521,600 (4.8)
Scotland	191 (9.2)	177 (8.5)	4,405,600 (8.4)
Northern Ireland	59 (2.8)	58 (2.8)	1,459,200 (2.8)
Total	2,084 (100)	2084 (100)	52,621,000 (100)

* Source: www.nomisweb.co.uk
